# The contribution of pre-stimulus neural oscillatory activity to spontaneous response time variability

**DOI:** 10.1016/j.neuroimage.2014.11.057

**Published:** 2015-02-15

**Authors:** Aline Bompas, Petroc Sumner, Suresh D. Muthumumaraswamy, Krish D. Singh, Iain D. Gilchrist

**Affiliations:** a Cardiff University Brain Research Imaging Centre (CUBRIC), School of Psychology, Cardiff University, Tower Building, Park Place, Cardiff CF10 3AT, UK; b INSERM, U1028, CNRS, UMR5292, Lyon Neuroscience Research Center, Brain Dynamics and Cognition Team, Hopital du Vinatier, 95 Boulevard Pinel, Bron, 69500, France; c School of Pharmacy and Psychology, Auckland University, Private Bag 92019, Auckland, New Zealand; d School of Experimental Psychology, University of Bristol, 12A Priory Road, Bristol BS7 8SW, UK

**Keywords:** Saccades, MEG, Phase, Amplitude, Decision, Free choice

## Abstract

Large variability between individual response times, even in identical conditions, is a ubiquitous property of animal behavior. However, the origins of this stochasticity and its relation to action decisions remain unclear. Here we focus on the state of the perception–action network in the pre-stimulus period and its influence on subsequent saccadic response time and choice in humans. We employ magnetoencephalography (MEG) and a correlational source reconstruction approach to identify the brain areas where pre-stimulus oscillatory activity predicted saccadic response time to visual targets. We find a relationship between future response time and pre-stimulus power, but not phase, in occipital (including V1), parietal, posterior cingulate and superior frontal cortices, consistently across alpha, beta and low gamma frequencies, each accounting for between 1 and 4% of the RT variance. Importantly, these correlations were not explained by deterministic sources of variance, such as experimental factors and trial history. Our results further suggest that occipital areas mainly reflect short-term (trial to trial) stochastic fluctuations, while the frontal contribution largely reflects longer-term effects such as fatigue or practice. Parietal areas reflect fluctuations at both time scales. We found no evidence of lateralization: these effects were indistinguishable in both hemispheres and for both saccade directions, and non-predictive of choice — a finding with fundamental consequences for models of action decision, where independent, not coupled, noise is normally assumed.

## Highlights

• We searched for oscillatory predictors of saccadic reaction times in humans.
• Pre-stimulus power at 5–45 Hz throughout dorsal pathway correlates with response time.
• These correlations mainly reflect spontaneous variance rather than deterministic factors.
• These correlations are dominated by different time scales across the brain.
• We found no evidence for a relationship between latency and phase of oscillations.

## Introduction

**Fig. 1:**
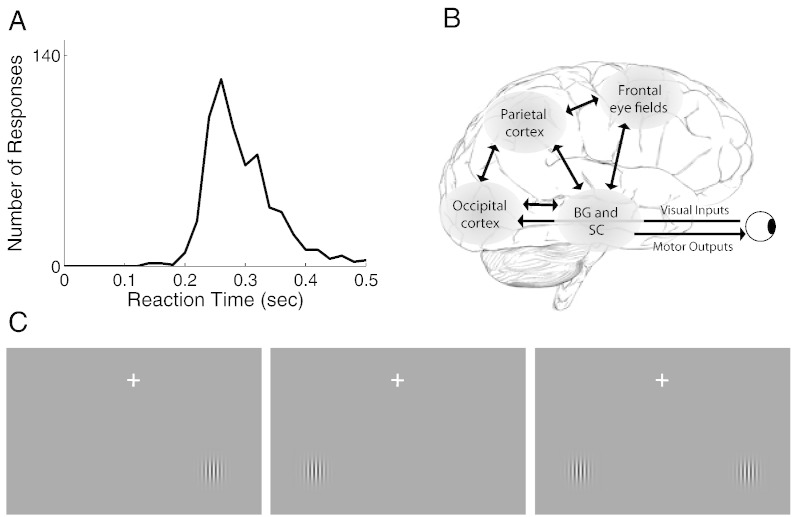
A. Reaction time distribution from one observer. B. A sketch of the sensory-motor saccade network, from where most of the oculomotor response variability must somehow arise. C. The simple tasks employed here. High-contrast Gabor patches were used as saccade targets. After a fixation period of between 3 and 4 s, the patch appeared in periphery either on the left or the right (single target trials) or on both sides simultaneously (choice trials).

The extent to which apparently random fluctuations in behavior are predictable is of fundamental theoretical and practical interest. The time taken to initiate even the most basic responses to highly salient stimulations typically varies four- to five-fold (Fig. 1A). It remains largely unknown why and when this variability occurs: how much is related to the experimental design (experimental factors, trial history, fatigue, practice etc.) and how much is stochastic; and to what extent it is predicted by pre-stimulus brain states. Although historically attributed to ‘noise’ (an unavoidable limitation of neural systems) and averaged away rather than investigated, variability is crucial to free an organism from predictable and stereotypic behavior. Indeed, models of sensorimotor decisions make an explicit link between variability in response time (RT) and variability in choice/decision (Brown and Heathcote, 2005; Carpenter, 2004; Rouder et al., 1998; Usher and McClelland, 2001).

After stimulus appearance, associations between neuronal activity and response time on each trial are clearly detectable both through monkey single unit and human whole-brain electrophysiology (Lee et al., 2010; Papadopoulou et al., 2010; Schall, 2001; Smyrnis et al., 2011). However, evidence for predicting response time variability from pre-stimulus neural markers is much less consistent, even though this time-period is increasingly thought to contain the seeds of the variance in electrophysiological responses to a stimulus (Arieli et al., 1996; Nikulin et al., 2007).

Our central interest lies in better understanding the sources of the large spontaneous variability observed in the speed of simple actions, such as an orienting response toward salient visual stimuli (Sumner, 2011). The present work focuses on characterizing and quantifying the contribution of pre-stimulus oscillatory activity to this variability. Existing literature directly related to this question only provides a fragmented, sometimes inconsistent, picture. In monkey, local field potentials suggest a complex pattern of positive and negative correlations of spontaneous alpha/beta fluctuations over dorsal areas with manual latency in a go–no go discrimination task (Zhang et al., 2008). Unfortunately, inconsistency across monkeys and the multi-component nature of the task make these data difficult to interpret. In humans, fluctuations in visuo-manual detection speed have been linked to increased fronto-parietal gamma power (Gonzalez Andino et al., 2005), while auditory-manual oddball detection speed has been linked to decreased fronto-centro-parietal gamma power (Reinhart et al., 2011). Saccadic speed has been linked to a slowly rising pre-stimulus EEG potential (Everling et al., 1997), and with the phase of alpha/beta oscillations either in occipital (Hamm et al., 2010) or frontocentral areas (Drewes and VanRullen, 2011). However, the analyses in EEG sensor space in Drewes and VanRullen (2011) and Everling et al. (1997) do not allow concurrent independent assessment of the contribution of each cortical area in the saccade generation network (Fig. 1B) to RT variability, while the absence of temporal jitter in the inter-trial-interval in Hamm et al. (2010) does not allow a distinction to be made between components related to motor response and target processing. Moreover, the actual predictive power of these markers has not been quantified to assess their contributions to predicting behavioral variability. Last, in most existing studies, trial-to-trial variance is assumed to represent spontaneous variance only, and the contribution from non-spontaneous sources (experimental conditions, trial order etc.) was not considered.

A related field of research relies on empirical modulations (rather than spontaneous variations) of pre-stimulus alpha power via sensory stimulation, and has suggested both positive (Kirschfeld, 2008) and negative (Del Percio et al., 2007) correlations with subsequent RT. However, beyond this apparent inconsistency, there is currently no evidence to tell us whether such empirical modulations in oscillatory activity should even be expected to have similar effects to spontaneous variability. Another related field of research focuses on the relationship between spontaneous pre-stimulus oscillatory activity and the visibility of near-threshold stimuli (Busch et al., 2009; Mathewson et al., 2009; van Dijk et al., 2008). However, the sources of visibility variation of near-threshold stimuli are unlikely to be identical to the sources of action variability to salient stimuli, as there has been long-term debate on the extent to which perception and action rely on dissociated neural pathways (see Milner and Goodale, 2008, for a review on this debate). There are certainly examples where factors with clear influence on RT do not affect perception (e.g. we respond slower to color changes than to luminance changes, but we do not perceive color changes as occurring later than luminance changes — Bompas and Sumner, 2008).

For all these reasons, to what extent MEG activity before stimulus onset predicts the spontaneous variance in action speed to clearly visible stimuli is still largely an open question. Our study aimed to resolve this question, by investigating both amplitude and phase of oscillatory activity, while also addressing related fundamental questions: Is variance correlated across the brain and across response options? How does such variance relate to choice outcome?

We therefore use a very simple task that maps a highly visible stimulus (no added noise and no perceptual uncertainty), presented alone or in pairs (free-choice trials) with temporal jitter, onto a highly practiced motor response (saccadic eye movements are among the quickest and most common sensorimotor actions we make, and the visuo-oculomotor network is well established, Fig. 1B), without further manipulation (Fig. 1C). We then searched for the MEG predictors of both saccadic reaction time in the no-choice trials and decision outcome in choice trials, using the pre-target period at which time the participants did not know which type of trial was about to appear. We use a variation of the beamformer source reconstruction approach to identify those areas where pre-stimulus amplitude predicted subsequent reaction time and quantify their contributions.

To characterize the contribution of spontaneous vs non-spontaneous sources, we compare our results when using, as regressors, the raw reaction times on each trial, or the reaction times corrected for main effects due to inter-trial-interval, experimental conditions or blockwise trends such as fatigue and practice. To further characterize the temporal dynamics and frequency spectra of this relationship, we reconstruct the activity at each step of cortical processing: in anatomical primary visual cortex (V1), intra-parietal sulcus (IPS), frontal eye field (FEF) and supplementary eye field (SEF). We then use the activity in V1 to assess correlations and independent contributions to RT across the brain. We also searched for predictors of choice outcome in two-target trials, and for a relationship between phase and reaction times.

## Materials and methods

### Observers

Twelve volunteers (4 female), with normal (or corrected to normal) vision participated (and received payment). The study received ethical approval from an independent local ethics board.

### Materials

The visual stimuli were presented on a Mitsubishi Diamond Pro 2070 monitor driven at 100 Hz, sitting outside the magnetically shielded room and viewed directly through a cut-away portal in the shield at a viewing distance of 215 cm. Participants were sitting in the dark; their head movements were restrained by a chin rest.

### Stimuli and procedure

Throughout the experiment the background of the display was set to mid-gray (25 cd/m^2^). At the start of each trial a small white fixation cross was presented (60 cd/m^2^; 0.1 × 0.1°). This was followed, after a non-aging fore period with a minimum of 3 s and a maximum of 4 s, by the presentation of the target display. With a non-aging fore period, the probability of target onset remains constant over time. The use of such a fore period results in the observer's expectation of when the target onset will occur remaining relatively constant throughout the fore period (Oswal et al., 2007).

There were three possible target displays (Fig. 1C): a single target in the lower left quadrant; a single target in the lower right; and two targets, one in the lower left and one in the lower right. Note that presenting stimuli in the lower quadrant rather than on the horizontal line means that the return saccade from the previous trial is not the same vector as either of the possible target directed saccades, thus reducing the relative importance of order effects (i.e. one source of deterministic variance) compared to spontaneous variability. All targets were a small Gabor patch presented close to 100% contrast with a spatial frequency of 4 c/° and an envelope with a standard deviation of 0.4°. Such stimuli are particularly salient targets for a saccadic response (Ludwig et al., 2004). After 800 ms the target display was followed by the fixation display for the next trial.

Trial types were randomly interleaved and participants were instructed to generate a saccade to a target as quickly as possible. On trials where two targets were presented, which we refer to as choice trials below, participants were allowed to saccade to either target, and instructed to avoid any conscious strategy, such as to alternate or balance the number of saccades to each side. 800 trials were collected from each participant in 4 successive sessions.

### Magnetoencephalography (MEG) hardware and recordings

Whole-head MEG recordings were sampled at 600 Hz (0–150 Hz band-pass), using the CTF-Omega 275 channel radial gradiometer system (VSM MedTech). Three sensors were turned off due to excessive noise. MEG data were manually screened trial-by-trial to reject all trials affected by artifacts such as head movements and muscle clenches. MEG/MRI coregistration was performed by placing fiduciary markers at fixed distances from anatomical landmarks identifiable in participants' anatomical MRIs (tragus, eye center). Fiduciary locations were verified afterwards using high-resolution digital photographs.

### Eye movement recording

Bipolar vertical and horizontal electrooculograms (EOG) were recorded at 600 Hz concurrently with the MEG signals. Signals were smoothed using a polynomial Savitzky–Golay filter with an order of 3 and a window size of 31 samples. Saccadic eye movement onset was automatically detected offline as the point in time where the absolute differential of the horizontal EOG signal was maximal, and visually checked in every trial. The vertical EOG traces were sometimes used to help clarify saccade onset. Trials were excluded when: the participant blinked or was not fixating well enough during the 1 s pre-stimulus baseline; the amplitude of the first saccade did not reach half the stimulus eccentricity; the saccade was directed away from the target on single target trials (directional errors); the latency was not between 75 and 500 ms or could not be determined with sufficient accuracy. Combining all these factors resulted in excluding 12% of the trials on average, with exclusion rates varying from 1% to 37% across observers depending on compliance and quality of the EOG signals.

### MR recordings

Structural MRI data for each participant were acquired on a 3-T General Electric HDx scanner using an eight-channel receive only head RF coil (Medical Devices). A 3D Fast Spoiled Gradient Recalled (FSPGR) scan was obtained in an oblique-axial orientation, with a 256 × 256 × 192 matrix at 1 mm isotropic voxel resolution (TR/TE = 7.9/3.0 ms; inversion time = 450 ms; flip angle = 20°).

### SAM source reconstruction

To reconstruct source activity we used synthetic aperture magnetometry (SAM) analysis (Robinson and Vrba, 1999; Van Veen et al., 1997; Vrba and Robinson, 2001). Our source reconstruction volumes were constrained to the individual brain volumes identified by FSL's Brain Extraction Tool and a multiple local spheres forward model (Huang et al., 1999) was used. For all analyses we used global covariance matrices and corresponding beamformer weight vectors.

**Fig. 2:**
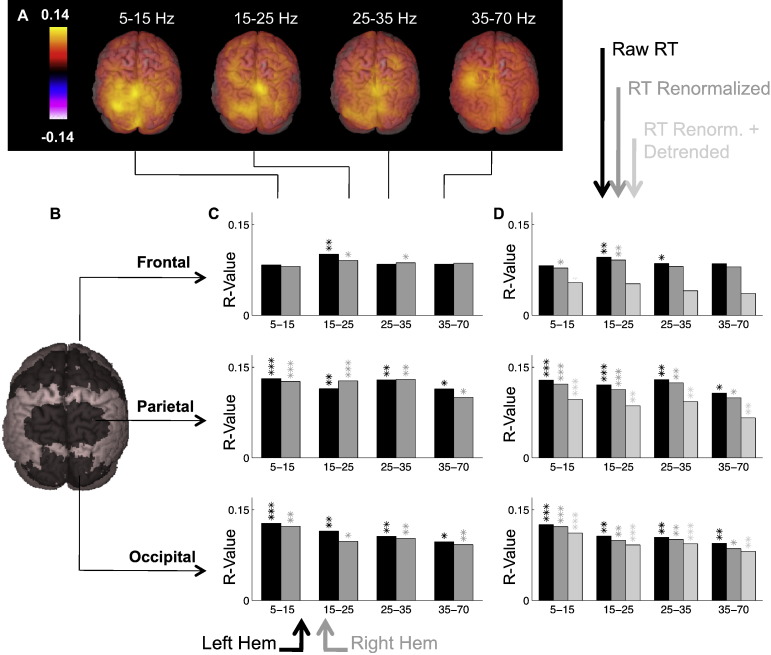
Saccade latency is significantly but weakly predicted by oscillatory power in the dorsal pathway during the 1 s pre-target period in alpha, low and high beta and low gamma. A. Each voxel represents the unthresholded Pearson coefficient (r value) between oscillatory power and saccade latency, averaged across left and right single target trials (which showed no difference). B. Correlations between oscillatory power and reaction times are displayed separately for three bilateral regions of interest (ROIs) along the dorsal pathway: a frontal region (Brodmann 6, including human FEF and SEF, top row), a parietal region (Brodmann 7, including superior parietal lobules and precuneus, middle row) and an occipital region (Brodmann 17 and 18, corresponding to visual areas V1 and V2, bottom row). C. Pearson correlation coefficient (peak r-value of the group-average within each ROI) between saccade latency and oscillatory power across trials during the 1 s pre-target period within 4 frequency bands, averaged across left and right single targets, for the left (black bars) and right (gray bars) hemispheres. D. Strength of correlations between oscillatory power and RT compared when using either raw RTs (black bars, averaged across the left and right hemispheres for clarity, i.e. the values correspond to the mean of the black and gray bars in panels C–D), RTs corrected for non-spontaneous variance only (dark gray) and RTs also corrected for linear trends within each block (light gray). Asterisks correspond to the peak p-value from the group statistics within each ROI, reflecting both the robustness of the effect and its spatial consistency across participants: */**/***p < 0.05/0.01/0.001 (corrected for multiple comparisons across voxels, ROIs and frequency bands using an FDR criterion).

For single left and right target trials separately, we performed a correlational SAM analysis. Since the whole visuo-oculomotor cortical network contains visually responsive cells, and oculomotor responses can be very fast, we restricted our analysis to the pre-stimulus baseline period, in order to avoid a confound of the stimulus and response locked activity. Beamformer weight vectors were calculated for each voxel in the source volume for time windows [− 1 s 0] and [− 0.2 s 0], in the following frequency bands: 5–15, 15–25, 25–35, 35–70 and 70–100 Hz. We then computed volumetric correlation images by linearly correlating, across trials, the saccadic latency of correct responses with the source amplitude activity in each frequency band during the trial baseline period (Figs. 2A–E). For choice trials, SAM analyses were performed for the same frequency bands and time window, contrasting all leftward with rightward choices.

### Isolation of truly spontaneous variance

**Fig. 3:**
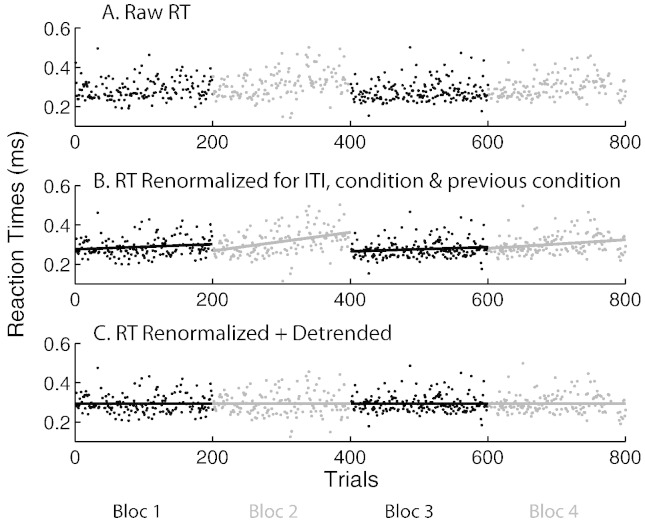
Illustration of the reaction time correction procedure of one participant. A. Raw reaction time on each trial across the 4 blocks. B. Reaction times after correction (renormalization) for the effects of: inter-trial-interval (ITI), current trial condition (single left, single right and bilateral) and preceding trial condition. Straight lines represent remaining blockwise linear trends across time. C. Reaction times further corrected (detrended) for these blockwise linear trends across time.

The above analyses were repeated with reaction times individually corrected for sources of variance directly linked to the experimental design (dark gray bars in Fig. 2D). We first corrected the RT for the linear effects of inter-trial-interval, and then for main effects of condition (left single, right single or choice) on the present trial and on the preceding trial, so that all nine combinations had the same mean RT (Figs. 3A–B). This correction thus included repetition and alternation effects (de Lange et al., 2013). Note that we could not also correct according to the choice made on the preceding choice trial (which would have produced 12 combinations) because for many participants there were not enough trials in every category to obtain reliable estimates of the means.

### Correction for long-term trends

A third type of regressor was computed, which further corrected for linear trends across time within each block (Fig. 3C), in order to account for fatigue and practice effects in direct relation to the design (light gray bars, Fig. 2D). We opted for the correction of all trends and effects, despite some being significant only on some participants or some conditions, rather than using a p < 0.05 arbitrary criterion. Since non-significant effects were small in amplitude, correcting for them only introduced minor changes to the regressors.

### Localization of V1, SEF, FEF and IPS

**Fig. 4:**
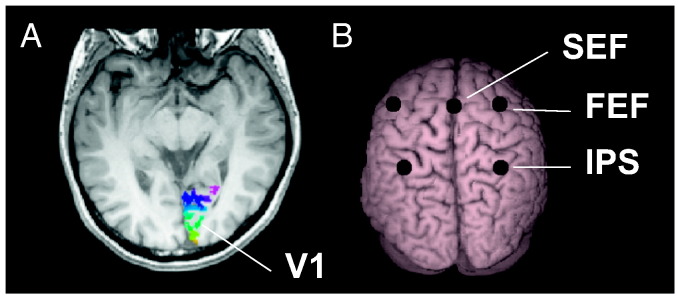
Anatomical V1, SEF, FEF and IPS used for defining virtual electrodes. A. Left V1 and right V1 were extracted individually and were each divided into 10 regions. In the center of each region was placed a virtual electrode, providing a sampling of MEG activity across V1. The extracted V1 regions on the right hemisphere are illustrated on one observer, color-coded from green (region corresponding to central visual field) to pink (peripheral). B. SEF, FEF and IPS on the template brain using the MNI coordinates provided by Mort et al. (2003) and Amiez and Petrides (2009), projected on the brain surface for illustration.

**Fig. 5:**
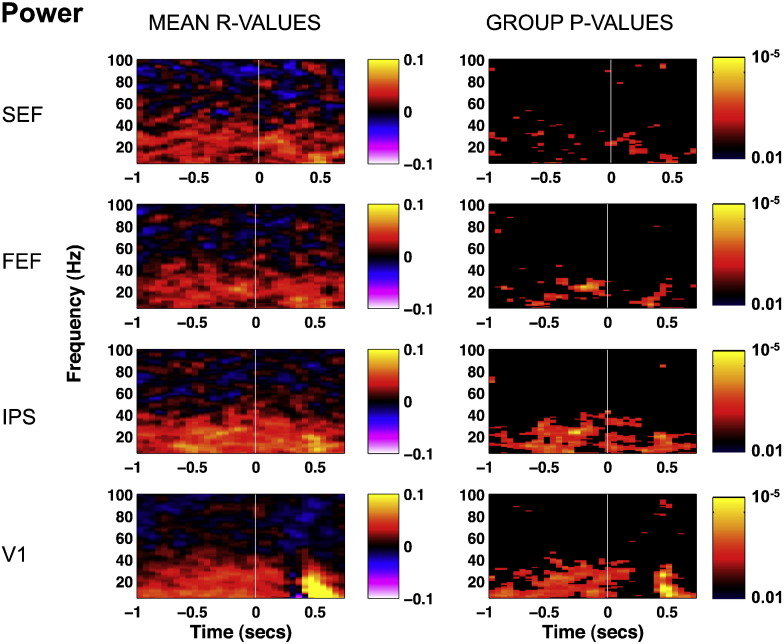
Time–frequency representation of the correlation coefficient (mean r-values across 12 observers, left column) between raw RT and oscillatory power and associated statistical significance (p-values, right column). p-Values were corrected for multiple comparisons and thresholded so that p-values below significance threshold appear in black, and p-values above threshold are color-coded according to their significance level. Time is averaged by bins of 50 ms. The FEF and IPS r-values are averaged across theleft and right electrodes. The V1 r-values are averaged across all 20 electrodes. The zero point indicates target onset. Spectral power from 5 to 40 Hz is positively correlated with RT during the whole 1-s baseline in V1 and IPS, and to a lesser extent in FEF and SEF.

**Fig. 6:**
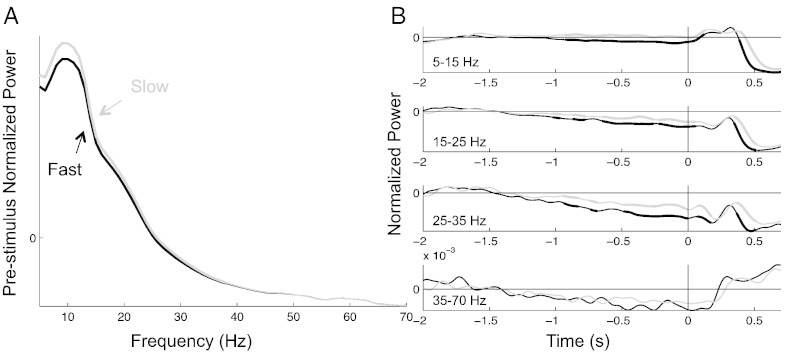
Power spectrum and time course compared for the fast (black) and slow (gray) halves of the reaction time distribution, mean of all V1 electrodes. Thick and thin lines indicate respectively significant and non-significant differences between fast and slow RTs at the group level after correction for multiple comparisons. A. Power spectrum during the 1-s pre-stimulus period, normalized so that the mean power from 5 to 70 Hz across all single target trials is equal to 0. B. Time course of oscillatory power in 4 frequency bands, normalized so that the mean power from − 2 to − 1 s across all single target trials equals zero.

**Fig. 7:**
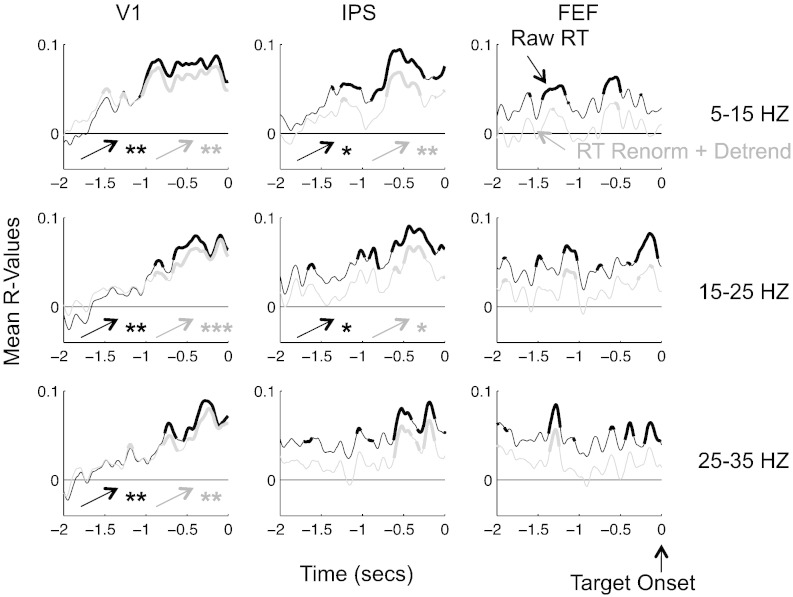
Time course of the correlation between oscillatory power and RT. Each line represents the group average of the r-values between RT and mean oscillatory power within an ROI and a frequency band, at each point in time between − 2 s and 0 with respect to target onset. Black and gray lines indicate the use of raw RT or RT renormalized and detrended as regressors. Thick portions indicate time bins where the r-values on the group were significantly above zero (after correction for multiple comparisons across ROIs, frequency bands, time points and regressor types). The asterisks next to the arrows indicate the significance of the increase of r-values across time: */**/*** p < 0.05/0.01/0.001 (corrected for multiple comparisons).

**Fig. 8:**
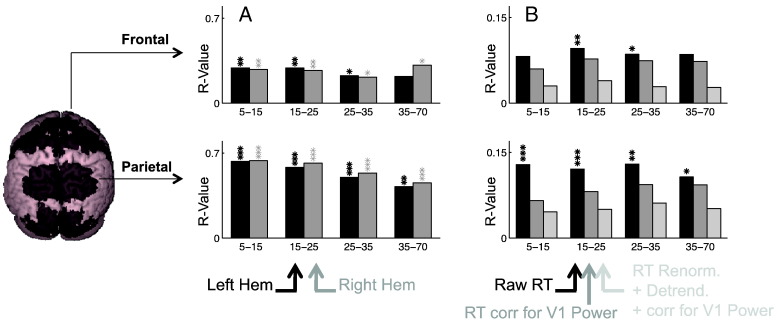
Correlation of oscillatory power across the dorsal pathway and estimated specific contributions from parietal and frontal areas. A. Correlation of pre-target oscillatory power between anatomical V1 and the parietal and frontal ROIs. Each bar represents the peak r-values of the correlation between oscillatory power within V1 (averaged across 10 virtual electrodes) and the 4 ROIs (left ROI in black and right ROI in gray), for the same frequency bands. B. Specific contributions of parietal and frontal regions to variability in saccadic latency. The graph compares the strength of correlations between oscillatory power and RT using either raw RTs (black bars, averaged across the left and right hemispheres, same values as black bars in Fig. 2D), RTs corrected for linear correlation with V1 power (dark gray bars) and RTs corrected for V1 power, deterministic effects and blockwise trends. Asterisks correspond to the peak p-value from the group statistics within each unilateral ROI (A) or within each bilateral ROI (B): */**/***p < 0.05/0.01/0.001 (corrected for multiple comparisons across voxels, ROIs and frequency bands).

**Fig. 9:**
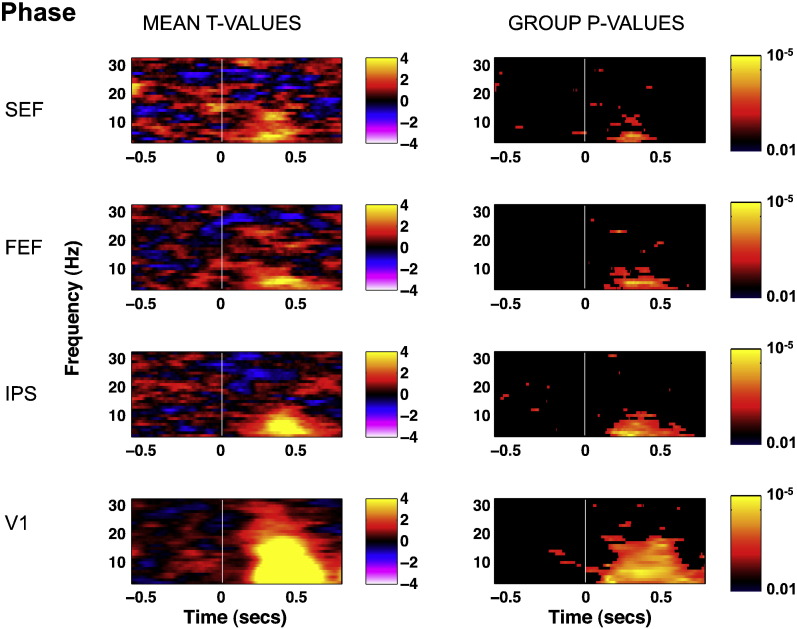
Results of the bootstrapping ITC analysis on RT quintiles across time and frequency. The left and right columns represent respectively the mean t-values across the 12 participants and 5 quintiles and the associated significance at the group level (p-values). p-Values were corrected for multiple comparisons and thresholded (black below threshold and color-coded above threshold). The FEF and IPS t-values are averaged across left and right electrodes. The V1 t-values are averaged across all 20 electrodes. The zero point indicates target onset.

To further characterize the time–frequency properties of the relationship between RT and oscillatory power, and to test for a relationship with phase, we reconstructed the MEG signal in multiple locations within the dorsal network. We wanted to avoid the circularity of defining virtual electrodes from the correlational beamformer analysis. We first tried to define functional localizers by contrasting post-stimulus and pre-stimulus time windows to reveal areas visually responsive or involved in saccade planning. However, probably because our task involved reflexive saccades to small peripheral visual targets, frontal and occipital contrasts were weak and not reliably present across individuals. In contrast, parietal activations were extended, making the selection of one peak for each individual difficult. We therefore used anatomically-driven locations. For primary visual cortex (V1), cortical reconstruction and volumetric segmentation for each participant were performed with the Freesurfer image analysis suite, which is documented and freely available (http://surfer.nmr.mgh.harvard.edu/). The automatic extraction and quantification of V1 were performed using a previously validated morphometric-based heuristic approach (Hinds et al., 2008). These mesh-based definitions of both left and right V1 were then split into 10 areas of equal length along an axis from central to peripheral vision in order to check for spatially selective effects (Fig. 4A). Parietal and frontal virtual electrodes were localized from the MNI coordinates provided in the literature (Fig. 4B): SEF [0 − 9 70.5] (Amiez and Petrides, 2009), left FEF [− 40 0 44], right FEF [28 − 6 50], left IPS [− 30 − 54 52] and right IPS [36 − 58 58] (Mort et al., 2003) and projected onto each individual. The center of each area was used as a virtual electrode (0–100 Hz bandpass) for the time–frequency (Figs. 5–8) and phase (Fig. 9) analyses.

### Time–frequency analysis of MEG power

To assess the relationship between oscillatory amplitude and saccade latency, we reconstructed the MEG signal on each trial for each virtual electrode. Virtual sensor weights were estimated using a 0–100 Hz optimal filter, and the 0–150 Hz signal was then projected through these weights. We calculated the analytic amplitude of the Hilbert transform between 5 and 100 Hz at 1 Hz frequency step intervals, using a ± 4 Hz Butterworth filter, from 2.7 s before to 0.7 s after target onset. We then post-decimated with a factor of 2. This was done for each trial type separately, left targets, right targets, choice trials with leftward saccade and choice trials with rightward saccades. For single target trials, we used the Pearson product–moment correlation coefficient to assess linear correlation across trials between the response latency and the power at each virtual electrode, each frequency and each 50 ms time window. The left column of Fig. 5 represents the mean across virtual electrodes (all 20 for V1, and left and right for IPS and FEF) and across left and right single target trials, since there was no evidence of spatially selective effects. For choice trials, we contrasted leftward with rightward choices. The same procedure was repeated on MEG channel to confirm our main result in sensor space (results are not shown).

### Inter-trial coherence analysis

Our analysis is copied from Drewes and VanRullen (2011). In brief, we sorted correct single target trials in each direction according to saccadic latency, split them into quintiles and calculated the amount of inter-trial coherence (ITC) within each quintile, using the phase component of the Hilbert transform described above. If the phase of ongoing oscillations has a direct effect on RT, then trials within a given quintile will have phases that are more similar than would be expected if there were no such relationship, yielding a greater ITC value. To assess the significance of any relationship between the phase of oscillations and RT in our data, we therefore calculated the ITC on non-sorted data, shuffling the trials randomly into quintiles and repeated this procedure 100 times to obtain the mean and SD of the null distribution, and thus derive t and p-values.

### Group analyses

Group analyses on SAM images were performed using permutation testing across participants (Nichols and Holmes, 2002; Singh et al., 2003) and p-values were corrected for multiple comparisons across voxels using the statistical omnibus. p-Values associated with the power and phase analyses from the virtual electrodes were obtained using a t-test comparing r-values (for amplitude) or t-values (for phase) from each participant to zero, independently for each frequency, time bin and ROI. All p-values were then corrected for multiple comparisons according to a standard false discovery rate calculation, taking into account frequency or frequency bands, ROIs, time bins within the time window of interest and regressor types, whenever relevant.

## Results

### Behavior

Saccade latency distributions were as expected. They typically showed a single mode around 250–300 ms, with hardly any saccadic responses before 150 ms or after 500 ms. Median saccade latency in single target trials ranged from 208 to 284 ms across participants, with a grand mean of 262 ms (264 ms on the left and 260 ms on the right, not significantly different). The mean of the standard deviation was 48 ms. Median latencies in free choice trials ranged from 220 to 310 ms, with a grand mean of 276 ms (276 ms for leftward choices and 278 ms on rightward choices, not different), and were significantly higher than in single target trials (mean difference 14 ms, t(11) = 5.03, p < 0.01). In choice trials, there was no overall bias toward one direction or the other: participants choose the left target on 51% of trials on average, but this proportion varied across individuals, ranging from 21 to 82%. Individual choice biases were correlated with latency differences between leftward and rightward saccades in single target trials, as predicted in accumulation models of decision (see Bompas et al., 2008, for a discussion).

ITI had a significant effect on RT for 5 subjects, but the direction was subject-specific, resulting in no overall tendency on the group (mean r = − 0.002, p = 0.95). Individually correcting for ITI accounted on average for 1.2% of variance (from 0.04 to 4% across individuals). Individually correcting for differences across all nine combinations of present and previous trial conditions (left, right, dual target) further reduced the variance by 7.9% (2.4 to 14.9% across individuals). Therefore together ITI, current and previous trial type accounted for 9% of the variance.

Blockwise differences and linear trends in each of the 4 blocks per participant were also block- and subject-specific; 20 of the 48 individual linear regressions (4 blocks and 12 subjects) were significant, but there were no overall tendencies (all p > 0.25). Arguably blockwise differences and trends are spontaneous, but they are at a larger time scale than trial-to-trial spontaneous fluctuations in brain states. Correcting for differences between, and trends within, each block for each individual mopped up another 8.6% of the variance on average (2.1 to 17% across subjects). Therefore, correcting for all these factors that do not reflect spontaneous short-term fluctuations accounted for 17% of the variance (8 to 25%).

### Contributions of broadband MEG power across the dorsal stream to saccadic RT

Areas showing significant correlation between MEG oscillatory power and RT were the whole of the posterior parietal lobe, extending into the brain to include the posterior cingulate cortex, most of the occipital lobe, the temporal–occipital junction and frontal areas corresponding to the anatomical definition of FEF (Fig. 2A). These correlations were consistently observed for frequency bands 5–15, 15–25, 25–35 and 35–70 Hz, but were not significant in the 70–100 Hz band. Note that oscillatory activity in the eye-balls was not predictive of subsequent RT, suggesting that the effect captured here does not merely reflect micro-saccades during fixation (Carl et al., 2012). Using a 200 ms or 1-s pre-stimulus time window gave the same results, but with increased power in the latter.

All these predictors of saccadic RT were nonspecific for target position or saccade direction. Nothing approached significance (all r < 0.1, p > 0.2) when subtracting the SAM images for left and right saccades, either with 1 s or 200 ms baseline. This interestingly contrasts with the lateralized effects observed in MEG during covert orienting of attention (Siegel et al., 2008), saccade preparation in delayed response paradigms (Hinkley et al., 2011; Van der Werf et al., 2008) and hand movement planning (de Lange et al., 2013; Donner et al., 2009).

To produce a figure summarizing our main result, we chose 6 regions of interest (ROIs) known to be involved in visuo-oculomotor decisions and comprising most of the areas revealed by the correlational SAM analysis (Fig. 2B). The 6 ROIs are: left and right Brodmann 17/18 (visual areas V1 and V2), left and right Brodmann 7 (including superior parietal lobules and precuneus) and left and right Brodmann 6 (including human FEF).

Peak r-values on each SAM image varied from − 0.03 to 0.34 across individuals, ROI and frequency band (hence a contribution to individual RT variance ranging from 0 to 12%). Each bar in Fig. 2C represents the peak value on the group-average of the parametric SAM images within one of these 6 large ROIs. Note that this constitutes a lower bound estimate of the contribution of these ROIs to RT variance because this measure is inherently sensitive to the spatial reliability at the voxel level across participants. The highest r-values were 0.13 (thus explaining around 1.7% of the variance in RT) and were found in parietal alpha and beta, closely followed by occipital alpha. An upper bound estimate is to consider the group-average of the individual peak r-values within each marker (defined as a combination of ROI and frequency band), leading to an estimated contribution from the occipital and parietal alpha and beta between 3 and 4% of the RT variance.

The strength of correlations was slightly reduced in frontal regions, as well as significance levels, possibly reflecting the reduced anatomical consistency of functional sources between individuals.

### Effect of RT corrections

Renormalizing RT for the effect of ITI and current and previous conditions did not affect the results (Fig. 2D, dark gray bars). This strongly suggests that correlations capture mainly endogenous variance. In contrast, detrending for blockwise trends reduced the strengths of correlation with oscillatory power consistently across all frequency bands, particularly in the frontal ROI, to a lesser extent in the parietal ROI and marginally in occipital areas (Fig. 2D, light gray bars). This suggests that these areas are differentially responsible for (or sensitive to) the various sources of variance that contribute to overall trial-to-trial variance (see section “Parietal and frontal contributions independent of V1”). In particular, frontal areas may reflect mainly long-term variance rather than trial-to-trial fluctuations.

### Power in V1, IPS, FEF and SEF and saccadic latency

The time–frequency analysis of single target trials in the 20 virtual electrodes in V1 and in IPS revealed that frequencies between 5 and 40 Hz during the whole 1 s baseline period were predictive of subsequent response time (Fig. 5). Similar but weaker patterns of correlation were observed in FEF and SEF. The predictive power was maximal in the alpha and beta ranges, but did not appear specific to any given narrow frequency bands. Rather, it consistently included the whole 5–40 Hz band in all participants. Beyond 40 Hz, correlations between amplitude and RT turned negative in some participants, while they remained positive in others, resulting overall in positive but not significant correlations. We replicated this analysis in sensor space across all sensors and obtained equivalent results.

A more traditional – though less powerful– way of visualizing data is provided in Fig. 6, where the pre-stimulus power oscillatory spectra and time courses in V1 are compared for the fast and slow halves of the RT distributions. Oscillatory power at all frequencies between 5 and 38 Hz was significantly higher in trials leading to long RT compared to short RT (Fig. 6A), peaking in the alpha range (with slow responses showing a 5% power increase compared to fast responses, t(11) = 5, p < 0.001). The effect built up over time, starting to be significant around − 1 s and reaching its maximum amplitude and statistical power around 100–200 ms before the target in all 3 lower frequency bands (Fig. 6B, all max t(11) > 5 and p < 0.001).

A more powerful way of assessing the time course of this effect is provided in Fig. 7. We observe a clear increase of correlation coefficients (r-values) over time from − 2 s to target onset in V1 and IPS, but not in FEF. In V1, mean r-values were about 0 at − 2 s and steadily increased until shortly before target onset, using either raw RTs or RTs corrected for longer-term trends. In contrast, r-values in IPS or FEF tended to be positive during the whole baseline period when using raw RTs. Using corrected RTs as regressors did lower the r-values throughout the time window (consistent with the results presented in Fig. 2B), bringing them down at the start of the time window, without affecting the time course of the effect.

Predictive power actually extends after target onset (Figs. 5 and 6), but here we focus our analysis on the baseline period. The very strong correlation at 500 ms post-target likely results from visual transients when the peripheral target is foveated, which only ever occur this late for long latency saccades (for shorter latency saccades the visual transient occurs earlier), consistent with the reversed correlation at this time.

No difference was observed between the 20 V1 virtual electrodes or between left and right target trials. In particular, this means that the virtual electrodes centered in the area of V1 containing receptive fields for the target or fixation point locations were not more predictive than the other virtual electrodes. A similar lack of spatial selectivity reported in the effect of temporal expectation on V1 activity using invasive recordings in monkey (Lima et al., 2011) suggests that this null finding may not simply reflect the limits of MEG spatial resolution but also the highly correlative nature of short-term fluctuations within the brain.

### Parietal and frontal power correlates with V1 power

We then investigated the independence of the different sources of variance identified in the correlational SAM analysis. We repeated the correlational SAM analysis using the oscillatory amplitude in V1 as regressors (instead of saccade latency). We did this analysis for the 5–15, 15–25, 25–35 and 35–70 Hz bands, using the mean V1 oscillatory power across all 20 electrodes in the same frequency band (Fig. 8A). This revealed high levels of correlation in oscillatory amplitude between anatomical V1 and the whole posterior half of the brain, including the occipital and parietal lobes, and up to FEF (Fig. 8A), as well as the cerebellum and the posterior temporal lobes (not represented), but not the rest of the cortex (lateral temporal or anterior frontal lobe).

### Parietal and frontal contributions independent of V1

To estimate independent contribution to RT variance from parietal and frontal areas, beyond that already accounted for by V1, we corrected RT for the linear trend of its association with mean V1 oscillatory power and used these corrected RT values as regressors in a new correlational SAM analysis. The analysis was repeated for the 5–15, 15–25, 25–35 and 35–70 Hz bands, using the mean V1 oscillatory power across all 20 electrodes in the same frequency band (Fig. 8B).

Correcting for the influence of V1 amplitude led to a reduction of mean r-values and associated significance levels in parietal and frontal areas. Predictive power remained significant across participants around anatomical FEF at 15–25 Hz and in the superior parietal lobule in the 15–25 and 25–35 Hz bands (all p < 0.05). Correcting for non-spontaneous effects and blockwise trends as well as V1 power (light gray bars, Fig. 8B) further reduced the mean r-values in both frontal and parietal ROIs. These results suggest that, although activity in the parietal and frontal areas is highly correlated across trials with activity in V1, parietal and frontal areas also make an independent contribution to RT variance, which mainly reflects longer-term trends, while short-term stochastic variance is mainly shared with V1.

### Phase and RT

Drewes and VanRullen (2011) reported an increased inter-trial coherence for trials leading to similar saccadic latencies than for trials randomly chosen independent of subsequent latency. This relationship was mainly observed in frontocentral areas between 11 and 17 Hz peaking 250 ms before target onset. In contrast, we see no evidence for such relationship between saccade latency and the phase of oscillations at any frequency between 5 and 35 Hz, any time point within the 0.6 s pre-stimulus period and in any considered area (V1, parietal, FEF and SEF, Fig. 9), except possibly for one small cluster (defined as at least two adjacent frequencies and time bins) in V1 (at 13–14Hz, − 0.240 to − 0.155 s, p = 0.004). Although this cluster matches in frequency and timing to that reported by Drewes and VanRullen, it is visible in V1, not in FEF or SEF and its strength is much reduced. Our analysis relies on about 530 trials per subjects when pooling across left and right single trials, which is comparable to Hamm et al. (2010), while Drewes and VanRullen used about 1800 trials per subject and per task. On the other hand, MEG with source reconstruction is known to offer better signal to noise ratio compared to sensor space analysis of EEG signal. In any case, rather than concluding that we have a non-replication of this previous finding, we will restrict our conclusion to stating that oscillatory power is a much stronger predictor of RT than phase in our dataset, since the former can be clearly identified on 500 trials, while the latter cannot.

### Predictors of choice

Although individual analyses often revealed predictors of subsequent choice when contrasting the baseline period preceding left and right choices, either with virtual electrodes or with the SAM analysis, these were not reliable at the group level. With the 1-s baseline period, group analysis identified 2 marginally significant clusters, in left middle occipital at 35–70 Hz, and in left inferior parietal lobule at 70–10 Hz (both p = 0.045). These clusters did not appear when we used the 200 ms baseline period and would not survive correction for multiple comparison across frequency bands and time windows.

We repeated this comparison in the − 100 to 80 ms time window (which most likely remains free from stimulus-related activity), and this analysis revealed other clusters for 15–25 Hz in the left post-central region, left middle frontal region and central precuneus (p-values = 0.02, uncorrected for frequency bands and time windows). We hypothesized that choice outcome would be more closely related to baseline state for shorter reaction times. However, replicating the analyses on the shorter half of the choice RT distribution also failed to reveal consistent predictors of choice. Furthermore, inter-trial coherence was not stronger across trials leading to the same response (left or right), than across trials randomly chosen independent of subsequent choice. The choice analyses relied on 266 trials, split between left and right choices, and we conclude that this was insufficient to successfully link subsequent choice to baseline oscillatory amplitude or phase.

## Discussion

### Summary of key results

1) The experiment presents clear evidence that broadband pre-stimulus oscillatory amplitude is positively correlated with response time to those stimuli, throughout, but restricted to the visuo-oculomotor cortical network, starting as early as V1, despite the stimuli being clearly supra-threshold. These correlations consistently extend from alpha to low-gamma.
2) These correlations capture between 1 and 4% of the RT variance and are not driven by deterministic sources of variance such as inter-trial-interval or stimulus side on the current and previous trials.
3) Although oscillatory amplitude is highly correlated between V1 and the rest of the network, parietal and frontal areas still make independent contributions: While correlations in occipital areas capture mainly short-term fluctuations, frontal and parietal contributions appear partly driven by longer-term sources of variance.
4) The results were indistinguishable for left and right responses, and left and right hemispheres; there was no hint of lateralization in the predictors of RT and no reliable predictor of chosen direction in free-choice trials.
5) We found no clear evidence for a relationship between latency and the phase of baseline oscillations. Our data thus suggest that oscillatory power is a stronger predictor of RT than phase.

### The whole dorsal pathway

The areas we identified perfectly match those known to be involved in visuo-oculomotor processing (e.g. Fig. 1B). Within this system, the cells in the occipital cortex are visually responsive and strongly interconnected with the parietal cortex, which has itself strong connections with frontal areas such as the frontal eye fields. As we move from occipital to parietal and frontal areas, the cells become more responsive to the task relevance of the particular visual stimuli and more tuned to the nature of the motor response required.

Our result thus suggests that response speed can be affected by both visual and premotor variability. The former is normally assumed to contribute mainly when the target is difficult to see or discriminate. For clearly visible unambiguous stimuli, as used here, motor variance is often assumed to dominate (Carpenter et al., 2009). However, we find that pre-target activity in striate cortex (V1) is clearly correlated to response time variability on a trial-by-trial basis, indicating that, even for supra threshold stimuli, an important component of the variability comes from differences in the state of basic perceptual processes. This finding is in line with a previous report that saccadic latency is predicted by the time of first spike elicited by the target onset in V1 (Lee et al., 2010) and implies that oscillatory power before stimulus appearance may influence (or reflect a process that influences) visual processing speed.

More fundamentally, oscillatory activity in V1 was strongly correlated with the rest of the dorsal pathway, suggesting that it may be the state of the whole sensorimotor system before stimulus onset that determines response readiness, rather than distinct perceptual or motor sources. This being said, correcting for oscillatory power in V1 did reveal independent contributions from parietal and frontal ROIs, even though these were not always spatially consistent enough across participants to reach significance at the group level.

The concurrent involvement of precuneus, inferior parietal and posterior cingulate cortices at all frequency bands from 5 to 70 Hz is possibly consistent with their being part of the default-mode network, in which BOLD activity has been found to correlate with cognitive performance in general, and reaction times in particular (Weissman et al., 2006). However, our analysis did not reveal the other areas in this network, such as inferior temporal or anterior medial prefrontal cortices.

### Broadband pre-stimulus oscillatory power

Accumulating evidence supports the idea that a large part of the variability in the behavior of neurons or individuals in response to a stimulus depends on the state of ongoing activity of the sensory and motor networks before the stimulus appears (Arieli et al., 1996; Mazaheri and Jensen, 2008; Nikulin et al., 2007). Previous attempts to relate pre-stimulus oscillatory power to response latency in visuomotor tasks have sometimes detected different subsets of the global pattern we find.

Reduced alpha amplitude around the occipital and/or parietal areas, whether spontaneous or resulting from attentional modulations, has been fairly consistently associated with better performance, either faster RT in easy tasks or improved perceptual discrimination for difficult-to-see stimuli (Kirschfeld, 2008; Klimesch et al., 1998; Makeig and Jung, 1995; Thut et al., 2006; van Dijk et al., 2008; Wyart and Tallon-Baudry, 2009; Zhang et al., 2008), though not always (Del Percio et al., 2007; Gonzalez Andino et al., 2005; Hamm et al., 2012). Increased alpha power has also been linked to better inhibition of distracting stimuli in a working memory task, and hence to faster RT to subsequent target (Bonnefond and Jensen, 2012). Moreover, reduced baseline alpha amplitude is thought to increase both the mean value of ongoing electrophysiological activity and the amplitude of subsequent visual evoked responses (Mazaheri and Jensen, 2008; Nikulin et al., 2007), both of which are linked with faster saccades (Everling et al., 1997; Papadopoulou et al., 2010; Smyrnis et al., 2011).

Beta amplitude in sensorimotor or frontal areas has also been associated with response times, although the direction of this association seems very much task and modality specific (Hamm et al., 2012; Linkenkaer-Hansen et al., 2004; Makeig and Jung, 1995; Perfetti et al., 2011; Zhang et al., 2008).

The amplitude of pre-stimulus gamma oscillations has been shown to be modulated by covert attention, although the direction of these associations seems to be design-specific (Fries et al., 2001; Siegel et al., 2008). Direct evidence that its spontaneous fluctuations are predictive of performance in visuomotor tasks is scarce and leads to unclear predictions. Hamm et al. (2012) observed that errors in an antisaccade task were associated with increased gamma power. On the one hand, this result can be seen as an inverse relationship between performance and gamma power, consistent with our result. On the other hand, since errors (prosaccades) are also faster than correct antisaccades, their result can also be thought to predict an inverse correlation between RT and gamma power. Gonzalez Andino et al. (2005) observed a *negative* correlation between RT and gamma power in a manual visuo-motor detection task, while Reinhart et al. (2011) reported a *positive* correlation in an auditory oddball task. Importantly, in these three previous studies, no significant correlation was reported in the dorsal pathway for lower frequencies (alpha or beta). Thus, contrary to *stimulus-induced* oscillations, where gamma shows opposite effects to alpha/beta, previous literature does not indicate that this reversal should also happen for spontaneous oscillatory power in relation to behavior. In our study, the direction of correlation was common across alpha, beta and low gamma (up to 45 Hz). Beyond 45 Hz though, we observed strong individual differences, with some participants showing strong positive correlations between gamma and lower frequencies, and some showing strong anticorrelations, resulting in no significant correlation overall.

In contrast to these previous studies, the signatures we report here are not restricted to one area or one frequency band. Rather, we observe consistent predictors of response time from alpha to low gamma (up to 45 Hz) and throughout the dorsal pathway. We suggest that the broad network is best revealed using a very simple task uncontaminated by other potential cognitive factors, combined with the parametric analysis approach we adopted.

It is possible that this consistent correlation from 5 to 45 Hz actually reflects a correlation with broadband power (Miller et al., 2014), which has recently been linked to local neurons' firing rates (Manning et al., 2009). This would offer the interesting possibility that low neuronal activity during baseline allows faster response to subsequent targets. However, we would then expect the correlations to extend up to higher frequencies, which we did not see. One possible reason for this is the reduced SNR of MEG signals when compared to intra-cranial recordings in these previous references, and the increasing relative contribution of non-brain signals such as instrumentation noise with increasing frequency.

### Quantifying contributions

Our aim was not only to identify neural markers in relation to behavioral variability, but also to quantify their predictive power. Our best predictors (amplitude of alpha and beta oscillations in parietal and occipital cortices) account, on average on the group, for 2% or 4% of the RT variance, depending on whether we pick the most reliable voxel at the group level or we allow the peak location to vary across participants within each ROI. The partial independence across ROIs makes it conceivable that predictive power could be improved by combining several markers and this will be the purpose of future work.

The literature does not offer estimates straightforwardly comparable to ours. To assess the relevance of a neural marker, most authors divide their data into categories (correct vs incorrect, fast vs slow RT, express saccades vs main mode) and only report the associated significance levels. In one study, the amplitude of pre-stimulus oscillations has been reported to account for 12% of the “response variability” in near-threshold visual discrimination (Busch et al., 2009). However, the authors mean something very different from what we are presently reporting: this 12% figure relates to a modulation in hit rate, rather than the proportion of variance explained.

A possible comparison of our figures is with respect to behavioral effects. For instance, in our study choice RTs to two-target trials are 18 ms longer on average than single target RTs, an effect highly significant (p < 0.001) and clearly expected from the literature (Leach and Carpenter, 2001). Using the same calculation as above, we can show that the variance of our RT vector is reduced by 2.8% if we renormalize for this effect, and thus this effect captures 2.8% of our overall behavioral variance. We may thus conclude that pre-stimulus amplitude of MEG oscillations accounts for RT variance to the same extent as any robust behavioral effect introducing a latency difference within the range of 15 to 20 ms.

### Spontaneous long-term and trial-to-trial fluctuations

When they are not simply ignored or averaged away, trial-to-trial fluctuations in performance or electrophysiological responses are usually considered to be i) endogenous and ii) independent across trials. However, all studies would necessarily involve sources of deterministic variance instrumental for the standard structure of experimental testing, such as inter-trial-interval or trial history. By correcting RTs for the estimated influence of such deterministic sources, we were able to safely conclude that the correlations we report capture truly endogenous variance.

Furthermore, brain activity is characterized by temporally correlated fluctuations that contribute to trial-to-trial variability but typically unfold at a larger time-scale (Hasegawa et al., 2000; Leopold et al., 2003; Menzer et al., 2010; Monto et al., 2008). By correcting RTs for the estimated effects of blockwise trends, we observed that correlations in frontal and parietal areas are partly driven by these long-term trends, while occipital contributions appear to capture mainly short-term fluctuations. Furthermore, the contribution from frontal areas that is *not* driven by longer-term trends appears to be mainly shared with V1.

### Yoked variability between left and right responses

Trial by trial variability plays a central role in contemporary models of perceptual-motor decision making (Bompas and Sumner, 2011; Brown and Heathcote, 2005; Carpenter et al., 2009; Ratcliff and Rouder, 1998; Rolls and Deco, 2011; Usher and McClelland, 2001). The neural origins of this ‘noise’ are assumed to account for both latency and outcome variability (choosing sometimes one option and sometimes another, or making correct versus error responses). At the core of these models is the idea that activity levels representing alternative possible responses race or interactively compete to reach a criterion threshold. Within these models, trial-to-trial noise in the baseline activity of the response options, the rate at which support for a particular response is accumulated and, potentially, the threshold level that leads to a response, combine to deliver latency variability and at the same time determine whether one response is generated over another.

Each of these factors could be reflected in or influenced by the pre-target activity we detect. Baseline and threshold variation would be expected to correspond to pre-stimulus activity in motor planning areas, while the pre-stimulus state of the perceptual system would affect the accumulation rate of visual ‘evidence’.

Crucially, however, to achieve variability in outcome (decision) the sources of ‘noise’ must either be independent between response options, or act in a directly push–pull manner, where any activity that favors one response will disfavor (or inhibit) the opposing or alternative response. In contrast to this assumption, all the pre-stimulus activity we detect to be associated with latency is positively yoked for both left and right responses. Such yoked sources of variability are irrelevant for choice/decision as they would not provide differential activity favoring one response over another.

Our results suggest that, in the absence of empirical manipulation introducing an overall bias, such as attention cues, unbalanced probability of appearance or reward, choice is mostly determined after stimulus onset. This result echoes the conclusions from previous studies showing distinct post-stimulus signatures in free choice compared to externally driven choices (Guggisberg et al., 2007; Pesaran et al., 2008). Although electrophysiological signals can be used for above chance decoding of covert intentions during free behavior (Jerbi et al., 2011), especially when participants are asked to withhold their response (Coe et al., 2002; Haynes et al., 2007), predicting free choice before it is explicitly made did not prove feasible with our design. Of course, it is possible that additional, spatially-specific, sources of noise are cryptic to MEG, but equally there may be other sources of yoked noise that are cryptic to MEG. What is clear is that the dominant portion of trial-to-trial pre-target activity variation visible to MEG is common to response alternatives.

### Phase and RT

Drewes and VanRullen (2011) reported a relationship between saccadic latency and the phase of EEG signals at frontocentral electrodes peaking 250 ms before target onset (50 ms before a fixed duration gap period), which they replicated on three different designs. We thus aimed to replicate their finding for frontocentral areas (expected to be close to our SEF electrode) and clarify the role of occipital and parietal areas. However, such relationship was not present in our data, in any considered area (V1, parietal, FEF and SEF, Fig. 9). This absence contrasts with the robust role of pre-stimulus alpha phase in visual perception for near-threshold stimuli (Busch et al., 2009; Dugue et al., 2011; Mathewson et al., 2009) and may be due to differences in task requirements or statistical power issues.
