# Corrigendum to “The contribution of pre-s timulus neural oscillatory activity to spontaneous response time variability” [Neuroimage 107 (2015) 34–45]

**DOI:** 10.1016/j.neuroimage.2015.04.002

**Published:** 2015-07-01

**Authors:** Aline Bompas, Petroc Sumner, Suresh D. Muthukumaraswamy, Krish D. Singh, Iain D. Gilchrist

**Affiliations:** a Cardiff University Brain Research Imaging Centre (CUBRIC), School of Psychology, Cardiff University, Tower Building, Park Place, Cardiff CF10 3AT, UK; b INSERM, U1028, CNRS, UMR5292, Lyon Neuroscience Research Center, Brain Dynamics and Cognition Team, Hopital du Vinatier, 95 Boulevard Pinel, Bron, 69500, France; c School of Pharmacy and Psychology, Auckland University, Private Bag 92019, Auckland, New Zealand; d School of Experimental Psychology, University of Bristol, 12A Priory Road, Bristol BS7 8SW, UK

(1) Page 34
Error: Aline Bompas ^a,b,⁎^, Petroc Sumner ^a^, Suresh D. Muthumumaraswamy ^a,c^, Krish D. Singh ^a^, Iain D. Gilchrist ^d^
  Corrigendum: Aline Bompas ^a,b,⁎^, Petroc Sumner ^a^, Suresh D. Muthukumaraswamy ^a,c^, Krish D. Singh ^a^, Iain D. Gilchrist ^d^
(2) Page 34
  Error: Rouder et al. 1998
  Corrigendum: Ratcliff and Rouder 1998
(3) Page 45
  Error: Rouder J.N., Ratcliff R., McKoon G. A neural network model of implicit memory in object recognition. J. Math. Psychol. 1998;42:489–489.
  Corrigendum: Ratcliff, R., Rouder, J.N., 1998. Modeling response times for two-choice decisions. Psychol. Sci. 9, 347–356.

